# Why did *European Radiology* reject my radiomic biomarker paper? How to correctly evaluate imaging biomarkers in a clinical setting

**DOI:** 10.1007/s00330-021-07971-1

**Published:** 2021-05-18

**Authors:** Steve Halligan, Yves Menu, Sue Mallett

**Affiliations:** 1 Centre for Medical Imaging, University College London UCL, 43-45 Foley Street, London, W1W 7TS UK; 2 Department of Diagnostic and Interventional Radiology, Saint Antoine Hospital, APHP-Sorbonne University, Paris, France

**Keywords:** Research design, Biomarkers, Publications, Radiomics, Peer review

## Abstract

**Abstract:**

This review explains in simple terms, accessible to the non-statistician, general principles regarding the correct research methods to develop and then evaluate imaging biomarkers in a clinical setting, including radiomic biomarkers. The distinction between diagnostic and prognostic biomarkers is made and emphasis placed on the need to assess clinical utility within the context of a multivariable model. Such models should not be restricted to imaging biomarkers and must include relevant disease and patient characteristics likely to be clinically useful. Biomarker utility is based on whether its addition to the basic clinical model improves diagnosis or prediction. Approaches to both model development and evaluation are explained and the need for adequate amounts of representative data stressed so as to avoid underpowering and overfitting. Advice is provided regarding how to report the research correctly.

**Key Points:**

• *Imaging biomarker research is common but methodological errors are encountered frequently that may mean the research is not clinically useful.*

• *The clinical utility of imaging biomarkers is best assessed by their additive effect on multivariable models based on clinical factors known to be important.*

• *The data used to develop such models should be sufficient for the number of variables investigated and the model should be evaluated, preferably using data unrelated to development.*

## Introduction

It has been said famously that, “Images are more than pictures, they are data” [1]. It is now relatively simple to apply algorithms to medical imaging that mine and extract multi-level quantitative data invisible to the naked eye, collectively termed “radiomics” [2]. A tsunami of research is investigating these and other “omics” currently, but a thin line divides such “Big Data” from “Bad Data”. The fact that medical imaging generates swathes of information is not, in-and-of-itself, sufficient reason to analyse it.

Radiomics are believed to be biomarkers, i.e. characteristics or measurements that reflect a physiological or pathological process [3]. Multiple radiomic or other imaging biomarkers are often investigated collectively, with the promise that they can predict important clinical events. A typical example claims that MRI predicts rectal cancer response to neoadjuvant therapy [4]. Increasingly, biomarkers are presented within a “model” that estimates whether an event will happen or not. However, the vast majority of these models are never used [5]. Why? Is it because radiologists resist new knowledge (unlikely!)? Is it because the models, although scientifically valid, are too complicated or time-consuming for daily practice? Or is it because they do not work? Informed consensus favours the latter: A recent viewpoint stated that publication of clinically useless prediction models was currently “exponential”, blaming easy availability of Big Data (including online image repositories) combined with inexpensive computational power to build models, but ignoring the methodological expertise necessary for proper development and evaluation [6]. A recent review noted that radiomic promise did not translate to daily practice, warning that radiological research must achieve “higher evidence levels” to avoid a reproducibility crisis such as that levelled at psychology [7].

As submission of such articles to *European Radiology* and other journals rises, as reviewers, we encounter the same methodological flaws repeatedly, meaning the model is unlikely to be useful. Publishing the model is therefore questionable. At the same time, we should encourage publication of early research where methodology is correct. To help improve the quality of such submissions to *European Radiology* (and the quality of the journal’s subsequent reviews), we describe in simple terms how to design a study that develops and evaluates a prognostic model of imaging biomarkers in a clinical setting. While some excellent articles on this topic exist [8], they require a reasonable grasp of statistics. We have omitted most mathematical detail, to focus on principles of good study design and analysis.

## Question 1: What am I trying to achieve and how should I proceed?

We hope researchers desire more than publication in an excellent journal like *European Radiology*, rather, discovery of a novel imaging biomarker adopted rapidly into clinical practice. We have noted that much radiomic research attempts to predict a clinical outcome, sometimes near future events like diagnosis, or later events, like survival. Predicting events of no clinical significance is irrelevant. For example, the clinical value of a model that predicts tumour genetics is minimal if the same information can be easily (and more accurately) obtained from a biopsy that is always performed routinely. So, such a model would go unused for rectal or gastric cancers. Because heterogeneity is common and clinically important, a case can be made that biopsy might not explore the whole tumour. A recent model attempted to predict Kirsten rat sarcoma (KRAS) mutation in rectal cancer from MRI “radiomics” [9]; however, accuracy was only “moderate”. In such cases, the research is acceptable where the authors clearly detail their route towards improvement. Ultimately, researchers should investigate a clinically useful question, one not answered better using other tests.

In the authors’ experience, while researchers might ask sensible questions, they may not answer them properly. Although the correct methods to identify and evaluate predictive biomarkers have been known for decades, they are commonly ignored. A 1994 article (27 years ago!) describing oncological predictors stated that correct methods were rare [10]. In particular, researchers focus on their “pet” biomarker(s) and ignore others already known to be clinically useful. Understanding the different phases of biomarker development is fundamental to avoid confounding pre-clinical, translational, and clinical stages [11] (Table 1). It is acceptable to research limited biomarkers for proof-of-concept, pre-clinical, exploratory studies. These determine whether a biomarker can be measured precisely and accurately, and how measurements vary by equipment, examiners, and patients. Recent initiatives address these issues of “technical validation” [12, 13]; unreproducible biomarkers are useless. Early research should determine appropriate diagnostic cut points for the biomarker (e.g. positivity threshold, operating point), so these are agreed before larger studies begin. Subsequent multi-centre pragmatic studies should focus on whether the biomarker(s) works in the intended clinical setting. Instead, investigating multiple different biomarker thresholds during an ongoing clinical study “moves the goal posts” and introduces bias because the dataset is being used for hypothesis generation and not biomarker evaluation.

**Table 1:** Descriptions of how study aims, subjects, and metrics vary for different phases of imaging biomarker assessment

Study phase	General study aim	Specific research question	Study subjects	Metrics measured
Preclinical	Radiomic biomarker discovery	Is the biomarker associated with the target pathology?	Patients with severe disease and with no disease. Phantoms	Technical validation (precision, repeatability, reproducibility, etc.)
Translational	Can the biomarker identify/predict disease?	Can the biomarker distinguish/predict diseased from normal patients?	Patients with severe disease and with no disease	Technical validation: (precision, repeatability, reproducibility, ROC AUC, etc.)
Early clinical: single-centre setting	Is the biomarker clinically useful?	Can the biomarker distinguish/predict all stages of the target disease and differentiate from patients without the disease (but who may have alternative diagnoses)?	Patients with all stages of the target disease. Patients seen in clinic but without the target disease	Diagnostic/predictive accuracy (sensitivity, specificity, detection rates, PPV, NPV, etc.)
				Diagnostic test impact (does the result impact on patient management?)
Late clinical: multi-centre setting	Is the biomarker generalisable and affordable?	Is the biomarker clinically useful and cost-effective in different centres and healthcare settings?	Representative patients of all who would receive biomarker test, with and without disease	Diagnostic/predictive accuracy
				Diagnostic test impact
				Cost-effectiveness

Clinicians are only moderately interested in single biomarkers unless they are exceptionally accurate and a reviewer or editor may regard such studies of little importance or even as “salami slicing”. It is common for multiple biomarkers to be assessed simultaneously. For example, researchers investigated whether DCE-MRI predicted therapeutic response in breast cancer [14]. Multiple MR parameters such as KTRANS (contrast transfer coefficient) were retrospectively identified and responders compared with non-responders: A significant difference was interpreted as “predictive”. Genetic factors were also compared, including hormone and HER2 receptor status. Such analyses present numerous *p* values comparing multiple different factors, with no clear relationship between them, and interpretation is difficult. Assessment of multiple biomarkers is facilitated if they are combined within a multivariable model that yields a single result (e.g. “response” or “no response”). Also, since diseases, patients, tests, and treatments are highly variable, a single factor is unlikely to be helpful when used alone. Such models are “prognostic”, which simply means prediction of a future event [15]. It follows that clinical assessment of an imaging biomarker is best achieved by incorporating it within a multivariable model, where its additive contribution to the overall result can be judged. For example, if we wished to assess whether KTRANS is clinically useful, we could develop a prognostic model to predict treatment response and then determine if adding KTRANS as a variable improves prediction significantly.

At this point, some self-criticism will help illustrate this common error: One of us assessed whether perfusion CT could predict rectal cancer metastasis by simply comparing baseline CT parameters between patients with and without subsequent metastases [16]; this is simply a measure of association, not prediction, and does not indicate clinical utility. While the paper was highly cited (N = 92), it did not influence clinical practice. This approach is only reasonable for early-stage development (Table 1).

## Question 2: What factors should be in my model?

Having established that imaging biomarkers should be examined within a multivariable model, the question then arises: what factors should the model contain? A model predicting breast cancer response but limited to DCE-MRI variables would be ignored by clinicians, whose clinical practice relies on tumour stage and hormone receptor status. Clinicians rarely treat patients on the basis of imaging alone and models must contain variables already proven useful. Consider appendicitis: The surgeon considers peritonism, pyrexia, tachycardia, and hypotension. Nevertheless, one model investigated sonographic appendiceal diameter alone [17]. Ignoring important non-radiological variables is a common mistake we often identify during review. This is probably because radiologists can obtain imaging data easily, but easy research is not good research. We are noticing similar issues with machine learning, where permissions necessary to use clinical data drive researchers towards more easily available factors.

While we encourage combining clinical and radiological variables, a balance must be struck between number and simplicity. Too many variables and the model is difficult to populate and use. Too few (especially omitting those deemed important by clinicians) and the model lacks face validity and will be ignored. Also, individual factors rarely contribute equally. The best contributes most utility, the second less so, the third even less. Accordingly, final models should retain factors “working hardest” and discard those not “pulling their weight”. Retained radiomics and other imaging biomarkers must contribute as effectively as clinical variables. An obvious advantage of assessment within a model is that the predictive strength of each individual variable is revealed by its additive contribution to the result.

Factors well established for a specific disease should always be included regardless of their statistical significance in a particular dataset. Clinicians will be surprised to learn that statisticians might ignore statistical significance when selecting variables. Rather, the recommended approach is to ask clinical experts to indicate factors believed important, only then adding less established factors. Unfortunately, the worst but most widespread approach is to select factors via their significance in univariable analysis, followed by retaining those that remain significant within the model [18, 19].

A recent article pleaded that models include all relevant clinical data before adding “omics” [20]. As proof, the authors developed a model for breast cancer survival that included TNM stage, age, receptor status, and grade. Adding omics (e.g. gene expression microarray data) failed to improve prediction. Indeed, omics only became useful if clinical data were *excluded*. Ultimately, the authors argued that novel biomarkers “may not be much more than surrogates for clinical data” [20]. Similarly, researchers investigating novel biomarkers to predict cardiovascular disease found they contributed little over and above simple clinical measurements [21].

It is worthwhile considering the term “biomarker”, defined by the USA National Institute of Health as “a characteristic that is objectively measured and evaluated as an indicator of normal biologic processes, pathogenic processes, or pharmacologic responses to a therapeutic intervention” [3]. While “biomarker” might imply a novel radiomic, genomic, proteomic measurement, in reality factors like age, sex, and smoking are strongly associated with “pathogenic processes”. Imagine a predictive model for colon cancer that omitted age and sex! It is imperative that models do not exclude factors simply because they are perceived as commonplace.

## Question 3: Do I have enough data to develop my radiomic model?

To develop a model, researchers need data from patients with and without the outcome of interest, e.g. patients who do and do not respond to treatment. They also need sufficient patients to avoid underpowering. Inadequate power is an almost ubiquitous shortcoming of submitted radiomic papers. A simple “rule of thumb” for prognostic research requires a minimum of ten events *per individual predictor investigated*, (noting that this is an initial step and additional sample size considerations remain important) [22–25]. Moreover, this rule applies to the *smaller* group, i.e. if the sample comprises 100 breast cancer patients, 30 of whom respond, then just *three* predictors should be investigated. “Clearly stated hypotheses are, unfortunately, rare in prognostic factor studies” [10], and researchers usually investigate too many factors. A study of CT to predict hepatic fibrosis developed a model using 53 patients, 16 without fibrosis [26]. As we have explained, those data are insufficient to examine more than one or two predictors yet 24 texture filter levels were investigated. In an even more extreme example, a study of 11 breast cancer patients studied 28 imaging biomarkers and 69 individual hypotheses yet, with just five non-responders, the researchers had insufficient data to examine even one [27].

Underpowered studies lead inevitably to false-positive (FP) results, i.e. type 1 errors: A systematic review of false discovery rates in radiological texture analysis found a mean chance of significant FP results of 76% [28]. Underpowering is widespread. Researchers must understand basic statistics: At a probability threshold of 0.05, the chance of a FP is 5%. This chance increases in line with the number of comparisons and is calculated easily: The chance of no FP is 0.95 per comparison. Thus, if we perform 10 hypothesis tests of independent biomarkers, the chance of no FP is 0.95^10^ = 0.6. Since the chance of no FP and *at least one* FP must = 1 (since one of these two events always occurs), then the latter is 0.40, i.e. there is a 40% FP chance from 10 hypothesis tests. These figures become increasingly alarming as comparisons increase: At 30 hypothesis tests, the chance is 79%, reaching 99% at 90 tests. Furthermore, this estimates the probability of *at least one* FP; far more are likely. Multiple statistically significant “omics” is usually “noise discovery” [29], and inadequate power means that most published significant findings may be false [30]. Researchers must have sufficient data to justify the number of factors investigated. If this is not possible, one approach is to reduce the number of individual factors investigated by using statistical methods that combine them: Principal component analysis (PCA) creates new combined variables and reduces dimensionality while preserving information. We direct readers to a review that details PCA methods for differing data, including where the number of extracted variables greatly exceeds the number of patients in the study (as is often the case for radiomics) [31].

## Question 4: How do I develop and evaluate my model?

So, assuming a sensible clinical question, the right combination of factors, sufficient patient events, then what next? The model is first “developed” (i.e. built) and then “evaluated” (i.e. tested). Many statisticians prefer “evaluate” to “validate” (which is widely used) because “validation” implies success. We describe basic principles but without detail because a qualified biostatistician should be involved and be a named author and statistical guarantor of the submitted work. Indeed, failure to involve a biostatistician probably explains the plethora of poor models [6].

Because they are inherently multivariable, models are usually developed using multivariable regression, with linear regression used for continuous outcomes, logistic regression for binary outcomes, Cox proportional hazards for time-to-event outcomes (notably survival), and additional methods such as Lasso and ridge regression. There are also methods that fit to classification rules, such as neural network and machine learning methods. Regression methods must be pre-planned carefully and considered along with clinical expertise and model objectives, including exactly how each factor is included, and whether selection methods such as “backward elimination” are appropriate [32]. Over-reliance on automatic selection by software/significance generally develops “overfitted” models, appropriate for the study dataset but inaccurate for new patients. Overfitting underpins why most models are never used; they do not work on new patients.

Model “discrimination” (how well does the model distinguish between patients with and without the outcome?) is often assessed early using the c-index or ROC AUC, although these do not evaluate clinical utility. Later evaluation establishes whether the model predicts the outcome accurately in representative patients [33], concentrating on how the model influences clinical trajectory. This, for example, may be expressed via proportions receiving different therapies with and without the model.

All models should be evaluated. We have noted that researchers usually have limited data. This forces researchers to both develop and evaluate their model using the same patients: “internal validation”. Several methods can be used, including bootstrapping, cross-validation, leave-out-one (“jackknifing”). These investigate the predictive impact of using slightly different patient combinations, sampled from the same study dataset. Bootstrapping is popular and uses the same number of patients but selected randomly with replacement so that, on average, around 60% of patients from the original dataset are included, with many represented multiple times. Typically, this is repeated between 100 and 2000 times. The impact of a few unusual patients is reduced and “shrinkage” of model coefficients can be completed so that it is not overfitted to a specific patient set. While understandable when studying rare conditions, internal validation is less appropriate for common diseases and presents a very weak challenge [34]. This is because patients used for development and evaluation originate from the same dataset, likely the same hospital, same clinics, examined with the same machines, measurement laboratories, etc. Internal validation only examines the effect of sampling error and ignores all other ways that patients might vary.

Evaluation is improved by using entirely new patients, preferably from different hospitals, with different staff, scanners, and data collection. Sometimes, as a compromise, a single dataset is divided into two separate samples, one for development (“training set”), and the other for evaluation (“test set”), often in a 2:1 ratio respectively [34]. However, this reduces development data and both samples still share a common origin. It is worth mentioning that researchers often split their data with good intent using randomisation but, for once, this is a trap! This is simply because randomisation aims to *minimise* differences between groups, thereby undermining the evaluation challenge. A better approach is to split data by time acquired, for example allocating the first two-thirds of recruits to development and the last third to evaluation; “temporal validation” [33]. Alternatives would be to split by imaging platform, for example allocating all patients from scanner A to development and all from scanner B to evaluation; a model that worked for only one CT vendor would be of little use. Or split by different clinics or geographic regions; physicians seeing the same disease recruit slightly different patient profiles. Or combinations of these tactics. Non-random splitting reduces overfitting and better compensates for this by shrinkage methods. Indeed, we reviewed a paper whose model used multicentre data, but rather than split development and evaluation data by centre, the researchers combined all available data and then generated two random samples, thereby missing a golden opportunity to evaluate their model properly.

Ideally, a model would work on patients anywhere, in different countries, attending different healthcare systems, examined using different staff, scanners, and measurement techniques. “Generalisability” or “transferability” describes this attribute and obviously requires evaluation with data from elsewhere: “external validation”. Most predictive models are never validated externally, and those that are often perform badly [35]. Shockingly, Chalkidou and co-workers found that only 3 of 15 radiomic studies used *any* validation (two of which were internal) [28]. We argue that any model submitted for publication that claims clinical utility must be evaluated, internally as a minimum and externally if possible.

## Question 5: Is the model equation presented in my paper? Is it easy to use and interpret?

During review, we usually encounter a large table displaying multiple factors, alongside odds/hazard ratios and *p* values. However, the final model equation recommended for clinical use should be presented so that others can evaluate it. Publication without the equation is rather like a recipe that omits the quantities of ingredients! The FRAX model has attracted considerable criticism because the equation is concealed [36], whereas the QRISK model was improved greatly by reviewers able to access the full data and equation [37]. Concealment underpins why many machine learning models are unlicenced. Clarity around the basis of prediction is fundamental so that healthcare staff and patients understand the evidence used to direct care [38]. Submitted papers must present detail sufficient for others to evaluate the model, or it cannot be used [39].

The equation is the final mathematical combination of variables and their weight. A simple example is the Nottingham Prognostic Index: Breast cancer survival = (0.2 × tumour diameter cm) + nodal stage + tumour grade [40]. Lower scores predict better survival. Models must be simple to use and interpret or they will be ignored, even when accurate. In reality, model presentation is complex and should consider the intended clinical setting (are computers available?), who will use it (clinicians or laypeople?), and when (during a fraught emergency or calm outpatient clinic?) [41]. Online calculators facilitate complex equations. Output must be easy to understand, using points, groups, or graphs. Nomograms are slightly more complex. The CRASH-2 model to predict death after trauma is an excellent example of a well-developed and evaluated model that is easy to use and interpret [42]: The patient’s age, Glasgow Coma Score, and systolic blood pressure are used alongside a simple chart that predicts the chance of death (in one of four categories, from < 6 to > 50%) depending on the economic status of the country where the trauma happened.

## Question 6: Is my research reported properly?

Model development and evaluation is complex but complete and precise descriptions of research methods are frequently missing [18, 43]. TRIPOD [44] and REMARK [45] are well-established guidelines for reporting prognostic research. TRIPOD describes 22 essential items that should be detailed in a prediction model study; a recent survey of radiomics publications found weak adherence with TRIPOD [46]. REMARK guidelines for tumour marker studies include a profile tool for reporting biomarker development and evaluation [47]. PROBAST judges risk-of-bias for prediction studies, comprising 20 signalling questions across participants, predictors, outcome, and analysis [48]. Authors should consult guidelines during study design rather than just before journal submission. This improves research quality and reporting greatly by flagging pivotal issues upfront, especially around how participants and their data are selected and analysed. It is also recognised increasingly that not only is it important to report research methods and findings, but that the “explainability” of any algorithm is also pivotal [49].

## Question 7: Defining my model—diagnostic, prognostic, predictive?

Whether a model is “diagnostic” or “prognostic” depends on the timing of the predicted outcome. In diagnostic models, the outcome is already present but unknown (e.g. disease), whereas prognostic models predict a future outcome. Defining “future” can be tricky: is tomorrow future? Broadly, diagnostic models use reference data to establish current outcomes whereas prognostic models use follow-up data to identify “true” future outcomes. Oncologists often state that “prognosis” predicts outcomes independent of treatment (e.g. TNM stage), whereas “prediction” evaluates outcomes after treatment [50]. Methodologists avoid specific nomenclature for healthcare models of treatment outcomes because they consider all participants receive “treatment” of some kind, even if that involves no active intervention.

## Question 8: Should I develop or update a model?

Models change over time as diagnosis and treatments change. It is more efficient to modify existing work rather than developing completely new models [51]. For example, researchers developed a model to predict outcome after brain trauma that included age, motor score, and pupil reaction, and then later incorporated midline shift from cranial CT to improve performance [52]. Models may need adjustment (updating) and recalibration (adjusting baseline events to local conditions) to account for different settings and/or patients. Models developed in one country may need updating for different healthcare settings. Unfortunately, most prognostic researchers develop new models rather than evaluate existing models; over 60 different models predict breast cancer outcomes [53]! This is especially unfortunate because evaluation studies are generally smaller and easier. Evaluating/updating existing models with new data is far more desirable and efficient than starting-from-scratch, and we need more radiological research on this topic. Combining older development data with new information will create more stable models. Updating might give different weightings to established factors, or add new factors [54].

## Question 9: Should I use systematic reviews and meta-analysis to identify potential biomarkers?

Biomarker research is commonly haphazard, inconsistent, and underpowered, with most appearing “promising” due to methodological error rather than intrinsic ability. However, if we assume genuinely promising biomarkers will appear in multiple studies, these may then be identified via systematic review. The review question must be framed carefully and precisely, and relevant populations, biomarkers, outcomes, and settings defined in advance. It is especially important to avoid muddling diagnostic and prognostic data by defining timings for biomarker and outcome measurements. Systematic reviews of prognostic studies are relatively novel, generating specific issues [55]. With sufficient primary data, prognostic effect estimates can be meta-analysed to obtain a signal of whether a predictor has genuine promise [55]. Extracted data will almost always be heterogenous as patients, study designs, and outcome definitions vary. Interpretation should therefore focus on significant associations with subsequent outcomes rather than the precise strength of that association. PROBAST should be used to appraise component studies [48, 56].

## Summary

Researchers seeking to properly develop and evaluate a radiomic biomarker in clinical practice face considerable hurdles, but good research is rarely easy. Publication of models that will never be used wastes research effort and must be discouraged. Furthermore, publication of inaccurate models risks harming patients. We hope our review will help researchers improve their methods when investigating radiomic and other imaging biomarkers in clinical contexts, and will also help reviewers when faced with these manuscripts. We have stressed that biomarkers should not be assessed without factors already known to be useful and that it is generally more desirable to evaluate/update existing models than develop new models. High-quality research is exceedingly unlikely without help from a co-author biostatistician, experienced in model development/evaluation.

## Abbreviations

DCE: Dynamic contrast enhanced
FP: False positive
HER: Human epidermal growth factor receptor
KRAS: Kirsten rat sarcoma
KTRANS: Contrast transfer coefficient
MRI: Magnetic resonance imaging
PROBAST: Prediction model risk of bias tool
ROC AUC: Area under the receiver operator characteristic curve.
TNM: Tumour, nodes, metastasis
TRIPOD: Transparent reporting of a multivariable prediction model for individual prognosis or diagnosis

## Declarations

### Guarantor

The scientific guarantor of this publication is Steve Halligan.

### Statistics and biometry

One of the authors (SM) is a medical statistician.

### Methodology

• Narrative review
